# Molecular Characterization of Primary Mediastinal Large B-Cell Lymphomas

**DOI:** 10.3390/cancers15194866

**Published:** 2023-10-06

**Authors:** Marie Donzel, Florian Pesce, Alexis Trecourt, Razika Groussel, Emmanuel Bachy, Hervé Ghesquières, Juliette Fontaine, Nazim Benzerdjeb, Claire Mauduit, Alexandra Traverse-Glehen

**Affiliations:** 1 Hospices Civils de Lyon, Institut de Pathologie Multisite, Hôpital Lyon Sud, 69310 Lyon, Francenazim.benzerdjeb@chu-lyon.fr (N.B.);; 2 UFR Claude Bernard Lyon 1, 69100 Villeurbanne, France; 3 Centre International de Recherche en Infectiologie (CIRI), UFR Lyon-1, Institut National de la Santé et de la Recherche Médicale (INSERM) U1111, Centre National de la Recherche Scientifique (CNRS), UMR5308, Ecole Normale Supérieure de Lyon, 69342 Lyon, France; 4 Institut Paoli-Calmettes, 13009 Marseille, France; 5 Centre Hospitalier de Roanne, 42300 Roanne, France; 6 Hospices Civils de Lyon, Service d’Hématologie, Hôpital Lyon Sud, 69310 Lyon, France; 7 Institut National de la Santé et de la Recherche Médicale, Centre Méditerranéen de Médecine Moléculaire (C3M), Unité 1065, Equipe 10, 06000 Nice, France

**Keywords:** primary mediastinal large B-cell lymphoma, PMBL, next generation sequencing, NGS, molecular pathology

## Abstract

**Simple Summary:**

The present study describes a primary mediastinal large B-cell lymphoma (PMBL) cohort on the morphological, immunohistochemical, and molecular levels, allowing to go deeper in the molecular characterization of PMBL. The mean age at diagnosis was 39 years (21–83), with a sex ratio of 0.88, and a predominance Ann Arbor stage II (67%). Most patients presented a non-germinal center phenotype (non-GC-B) using the Hans algorithm (88%). CD30 was expressed in 88% of cases; with a partial and heterogeneous (67%) or intense and diffuse (20%) expression. CD23 was expressed in 75% of cases with a focal (8%), partial (45%), or diffuse (22%) expression. CIITA breaks were observed in 35% of cases. None of the cases displayed BCL2 rearrangement. The most frequent mutations were: *SOCS1* (91%), *TNFAIP3* (54.5%), *ITPKB* (51.5%), *GNA13* (48.5%), *CD58* (36.4%), *B2M* (36.4%), *STAT6* (33.3%), *ARID1A* (30.3%), *XPO1* (27.3%), *CIITA* (24%), and *NFKBIE* (24%). These data also provide pathologists with daily routine tools and reinforce the interest in an integrated histomolecular diagnosis to allow precision diagnosis as early as possible and to adapt the therapeutic strategy.

**Abstract:**

Since the description of primary mediastinal large B-cell lymphoma (PMBL) as a distinct entity from diffuse large B-cell lymphomas (DLBCL), numerous studies have made it possible to improve their definition. Despite this, this differential diagnosis can be challenging in daily practice. However, in some centers, PMBL may be treated according to a particular regimen, distinct from those used in DLBCL, emphasizing the importance of accurate identification at diagnosis. This study aimed to describe the histological and molecular characteristics of PMBL to improve the accuracy of their diagnosis. Forty-nine cases of PMBL were retrospectively retrieved. The mean age at diagnosis was 39 years (21–83), with a sex ratio of 0.88. All cases presented a fibrous background with diffuse growth of intermediate to large cells with an eosinophil (26/49, 53%) or retracted cytoplasm (23/49, 47%). “Hodgkin-like” cells were observed in 65% of cases (32/49, 65%). The phenotype was: BCL6+ (47/49, 96%), MUM1+ (40/49, 82%), CD30+ (43/49, 88%), and CD23+ (37/49, 75%). Genomic DNAs were tested by next generation sequencing of 33 cases using a custom design panel. Pathogenic variants were found in all cases. The most frequent mutations were: *SOCS1* (30/33, 91%), *TNFAIP3* (18/33, 54.5%), *ITPKB* (17/33, 51.5%), *GNA13* (16/33, 48.5%), *CD58* (12/33, 36.4%), *B2M* (12/33; 36.4%), *STAT6* (11/33, 33.3%) as well as *ARID1A* (10/33, 30.3%), *XPO1* (9/33, 27.3%), *CIITA* (8/33, 24%), and *NFKBIE* (8/33, 24%). The present study describes a PMBL cohort on morphological, immunohistochemical, and molecular levels to provide pathologists with daily routine tools. These data also reinforce interest in an integrated histomolecular diagnosis to allow a precision diagnosis as early as possible.

## 1. Introduction

Diffuse large B-cell lymphomas (DLBCL) represent about one-third of histologically diagnosed lymphomas [1,2]. It is a very heterogeneous group, including morphologically, phenotypically, and clinically distinct entities. With the development of immunohistochemistry and molecular biology, understanding these pathologies has evolved considerably, and DLBCLs have undergone a critical dismemberment, enabling the emergence of many new entities. Appearing among them is primary mediastinal large B-cell lymphoma (PMBL), which has been described as a separate entity since the 2001 World Health Organization (WHO) classification of lymphoid tumors [3,4]. Before that, this pathology was only considered to be a subtype of DLBCL occurring in the mediastinum. But with scientific advances, many observations have allowed us to distinguish it from other large cell B lymphomas.

Of all lymphomas, PMBL represents about 2 to 3% of non-Hodgkin’s lymphomas and 10% of all diffuse B lymphomas [3]. Typically, this lymphoma affects the mediastinum in young adults (third or fourth decade of life) with a female predominance [3], which may cause local symptoms such as superior vena cava syndrome, cough, or dyspnea. Extra-nodal involvement is rare.

Since PMBLs have been described as a separate entity, many studies have been conducted to characterize these tumors at the molecular level. Concerning their cellular origin, PMBLs have been identified as originating from thymic B cells, particularly because of their CD23 expression (coming from mature B cells circulating through peripheral blood or derived from early T-cell progenitors that colonize the thymus from intrauterine life and switch to the B lineage) [5,6]. They present maintenance of the B-program with an intense and homogeneous expression of B-cell markers (CD20, CD79a) [7] or transcription factors (BOB1 and OCT2) [8]. Immunoglobulin gene rearrangement is found in 35 to 65% [7,9]. With the appearance of classifications based on cell-of-origin, thanks to transcriptomic data, it appears that this entity, despite its non-GCB phenotype, is closer to the germinal center DLBCL (GC-DLBCL), instead of activated B-cell DLBCL (ABC-DLBCL), particularly when using reverse transcriptase multiplex ligation-dependent probe amplification (RT-MLPA) [10]. However, molecular sequencing data have demonstrated that PMBL were neither attached to the group of GC-DLBCL nor ABC-DLBCL but seemed related to classical Hodgkin Lymphomas (CHL) [11,12,13,14]. It has recently been shown that PMBL and CHL constitute the opposite ends of the same lesion spectrum, in the middle of which lies the diversity of grey zone lymphomas [15,16].

These entities are treated according to a particular regimen, different from those used in CHL, and often more intensive than those used in DLBCL, emphasizing the importance of distinguishing them from these differential diagnoses at diagnosis. Admitted treatments are DA-EPOCH-R (dose-adjusted etoposide, prednisone, vincristine, cyclophosphamide, doxorubicin, and rituximab), R-ACVBP (rituximab, doxorubicin, cyclophosphamide, vindesine, bleomycin, and prednisone), or in R-CHOP, 14 or 21 chemotherapy (rituximab, cyclophosphamide, doxorubicin, vincristine, and prednisone) [17], sometimes associated with consolidation radiotherapy with good responses [17,18]. However, in relapsed/refractory cases, the prognosis remains poor, with few therapeutic weapons, including single-agent PD-1 inhibitor pembrolizumab [17,19], which may be combined with the CD30 antibody drug-conjugate brentuximab vedotin (BV) [18]. In advanced forms, anti-CD19 chimeric antigen receptor T-cell (CAR T-cell) therapy has also demonstrated results [18]. However, it remains the case that prognostic markers are lacking in PMBL. In the era of targeted therapies, it is crucial to differentiate PMBL from other entities, particularly DLBCL, which can invade the mediastinum or initially present as a mediastinal mass.

Moreover, in real life, the differential diagnosis is not always easy on a mediastinal mass. DLBCL may present as a mediastinal localization and may express CD23 in 10% of cases [20,21] or CD30 in 30% of patients [22]. In addition, clinical information is only sometimes available or complete during pathology diagnosis.

The aim of this study was, therefore, to see if the mutational profile could help in the diagnosis and be discriminating, as in other entities, by correlating morphological aspects and molecular data from NGS (next generation sequencing) to refine the diagnostic criteria and to open the way to innovative therapies.

## 2. Materials and Methods

### 2.1. Selection of Cases

Primitive cases of PMBL were retrospectively retrieved from the Pathology Department of the Lyon-Sud University Hospital from January 2018 to December 2020. The diagnoses were established following the 2022 WHO (World Health Organization) and ICC (International Consensus Classification) criteria. Cases were reviewed by expert hematopathologists (ATG, JH, MD) of the national Lymphopath network, which reviews every new diagnosis of lymphoma in France [1].

### 2.2. Clinicopathological Characteristics

Data including age, sex, clinical presentation (B signs, dyspnea, cough, thoracic pain, presence of superior vena cava syndrome (SVCS), presence of lymphadenopathies, the size of the tumor, results of the extensive assessment, received treatments, and evolution) were collected.

### 2.3. Immunochemistry and Study

The protocol and antibodies are detailed in Supplementary Materials and Methods and Table S1. Most markers, including CD20, CD30, CD10, CD15, BCL6, BCL2, CD23, and MUM1, were assessed qualitatively (focal, partial, diffuse, or negative) with a predefined scoring system. Labeling was considered “diffuse” if more than 75% of cells were labeled, “partial” if 30 to 75% of cells were labeled, and “focal” if less than 30% of cells were stained. This scoring system was adapted for BCL2 and CMYC. BCL2 when there were ≥50% of stained cells, with a partial staining if 50 to 75% of cells were stained, or diffuse staining if >75% of cells were labeled [23]. CMYC was considered positive when >40% of the tumor cells had nuclear staining, with a partial staining if 40 to 75% of cells were stained or a diffuse staining if >75% of cells were labeled [23]. CD10, BCL6, and MUM1 were defined as positive if at least 30% of neoplastic cells were stained, according to the Hans algorithm for DLBCL [23]. Epstein–Barr virus (EBV) was assessed in tumor tissue using an in situ hybridization technique (ISH), targeting EBER -1 and -2. A control was present on each slide, represented by an EBV-positive diffuse large B-cell lymphoma, not otherwise specified (NOS). Cases with less than 10% of small stained cells were considered “bystander cells” [24]. Results of EBER-ISH were considered as diffusely positive if they were at least 80% of stained tumoral cells (by analogy with large cell B lymphoma), partial if EBV+ cells represented 10 to 80% of the infiltrate, and negative if stained cells represented less than 10% of the infiltrate [24,25,26] and after confirmation using EBV-latent membrane protein 1 (LMP1) immunohistochemistry. The proliferative index was evaluated as the percentage of Ki-67-positive cells relative to the total lymphomatous population.

### 2.4. Fluorescence In Situ Hybridization (FISH)

The results of fluorescence in situ hybridization for MYC, BCL2, BCL6, and CIITA were collected. Fluorescence in situ hybridization (FISH) was performed as previously published [27], using the BCL2/18q21, MYC/8q24, BCL6/3q27 (ZytoLight; ZytoVisionTM; 27572; Bremerhaven; Germany) and *CIITA*/16p1313 (Empire Genomics; 14221, New York, NY, USA) break-apart probes. At least two physicians carried out the interpretation. A hundred cells were counted per probe, and a cut-off of 10% was used to retain a break.

### 2.5. Targeted Next-Generation Sequencing Analysis (TNGS)

Only cases with available FFPE material were included in the TNGS analysis.

Genomic DNAs were tested by TNGS using a custom design panel [27,28,29] of 39 genes (*ARID1A*, *B2M*, *BTG1*, *CCND3*, *CD58*, *CDKN2A*, *CDKN2B*, *CIITA*, *CREBBP*, *CXCR4*, *EP300*, *FOXO1*, *GNA13*, *ID3*, *IRF4*, *ITPKB*, *JAK3*, *KLF2*, *MEF2B*, *MFHAS1*, *MYC*, *MYD88*, *NFKBIE*, *NOTCH1*, *NOTCH2*, *PIM1*, *PRDM1*, *PTPRD*, *RHOA*, *SOCS1*, *STAT3*, *STAT6*, *TET2*, *TNFAIP3*, *TNFRSF14*, *TP53*, *TRAF2*, *TRAF3*, and *XPO1*), determined by a consensus of French Lysa experts to capture the main driver mutations of lymphomas [29,30]. Libraries were prepared with the Sophia Genetics (Rolle, Switzerland) or QIAseq (Qiagen, Courtaboeuf, France) technology starting from FFPE samples, according to the manufacturer’s recommendations. Samples were sequenced on a Nextseq 500 device (Illumina, San Diego, CA, USA) with a mean sequencing depth of 4432.63 (ranging from 257 to 27,313) after demultiplexing, alignment, and variant calling were performed with a previously described pipeline [31]. A VAF ≥ 5% cut-off was used to avoid most of the formalin fixation artifacts, according to the recommendation of the Association for Molecular Pathology and College of American Pathologists [32]. The interpretation was performed with the SOPHiA GeneticsTM DDM v4 pipeline with OncoPortal v1.1. Mutations retained in the report were class 3, 4, or 5 only (uncertain clinical significance, likely pathogenic and pathogenic, respectively).

### 2.6. Statistical Analysis

Statistical analysis has been performed using the Medistica, pvalue.io, a graphic user interface to the R statistical analysis software for scientific medical publications, 2019–2022. Available on: https://www.pvalue.io, performing Fisher’s exact test or χ2 test.

## 3. Results

A total of 49 specimens with the histological diagnostic of PMBL were identified.

### 3.1. Epidemiological Characteristics

Epidemiologic and clinical data are summarized in Table 1. Most of the clinical information was missing for the external cases received in our laboratory for expert review, in particular stage and follow-up.

The mean age at diagnosis was 39 years (21 to 83 years). The sex ratio was 0.88. A mediastinal mass was present in all cases. Patients presented either a mediastinal mass, isolated or associated with supra diaphragmatic lymph nodes, corresponding to Ann Arbor stage II (22/33, 67%), or a systemic disease, with subdiaphragmatic lymph node or distant organ involvement corresponding to Ann Arbor stages III or IV (11/33, 33%). The most represented first-line treatment in the present study was R-ACVBP (19/25, 76%), followed by a subsequent sequential consolidation (two cycles of methotrexate, four cycles of rituximab with etoposide and ifosfamide and two cycles of cytarabine) (17/19, 90%), or autologous stem cell transplant in two cases (2/19, 10%). Of the 25 patients for whom follow-up data were available, 84% (21/25, 84%) had complete remission after the first line, and four relapsed within the first year (4/25, 14%) after a mean follow-up time of 35 months (from 12 to 57 months). Relapsing patients were treated using CAR T-cells (3/4) or brentuximab vedotin (1/4).

### 3.2. Histological Aspect

**Figure 1:**
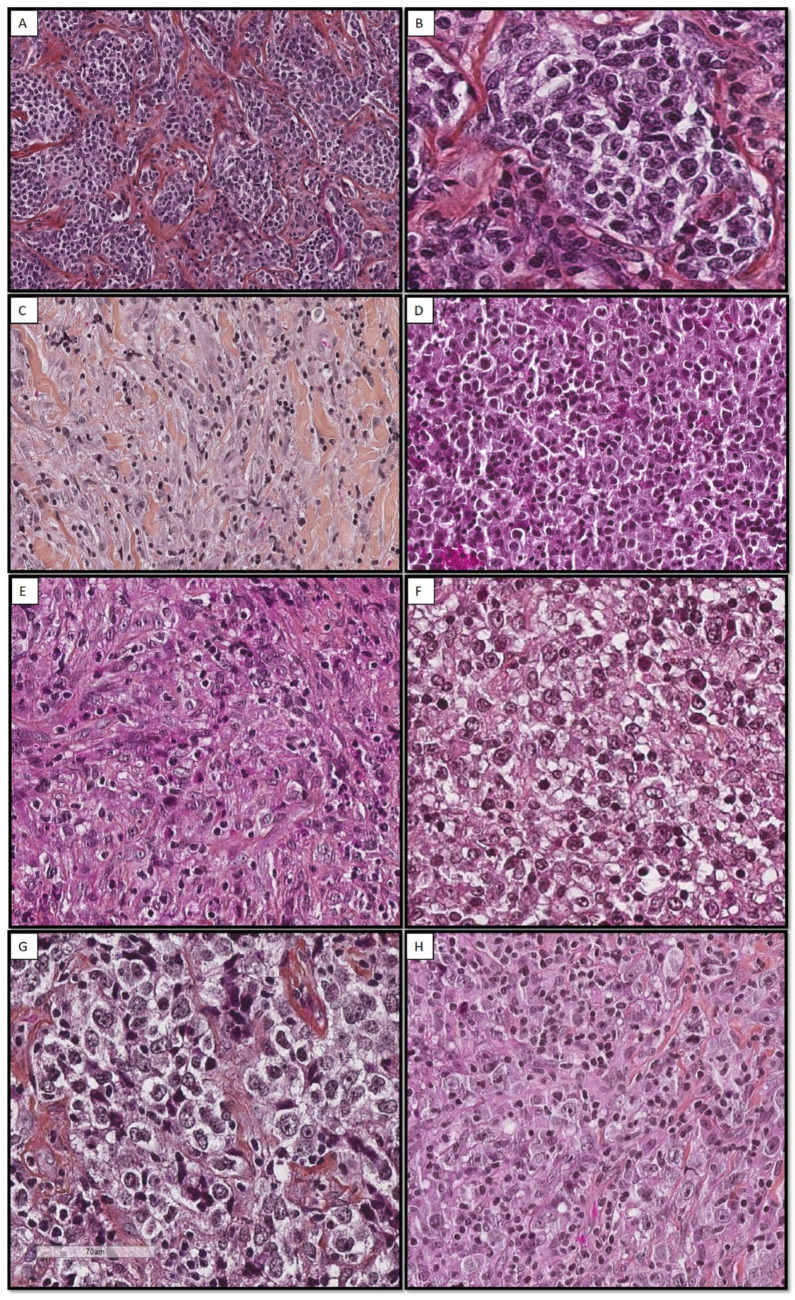
Histological aspects of eight cases of PMBL. These pictures illustrate the morphological aspect of PMBL. These lymphomas mostly have a diffuse architecture (**C**–**F**) but frequently display prominent sclerosis (**A**–**C**,**G**), which is sometimes responsible for a vaguely nodular architecture (**A**,**B**,**G**). Lymphoid cells are intermediate to large. They present round (**B**,**D**,**F**,**G**) or fusiform nuclei (**C**,**E**), surrounded by an abundant retracted (**B**,**F**,**G**) or eosinophil (**E**,**H**) cytoplasm. Some cases contain Hodgkin-like cells (**E**,**G**), focally associated with eosinophils (**E**).

The histological diagnosis was performed on mediastinal (33/49, 67%) or nodal (12/49, 24%) core needle biopsy, nodal resection (3/49, 6%), or bone biopsy (1/49, 2%). All cases presented a fibrous background, consisting of focal (9/49, 18%), moderate (24/49, 49%), or extensive (16/49, 33%) fibrosis, responsible for cell crushing artifacts that interfered with the analysis. Necrotic foci were observed in 30% of cases (14/49, 29%). Cases exhibit diffuse growth patterns (38/49, 78%), and some displayed a vaguely nodular architecture due to fibrous changes surrounding some clusters of lymphomatous cells (11/49, 22%). Lymphoma cells were large and had eosinophilic (26/49, 53%) or optically empty/retracted (23/49, 47%) cytoplasm (Figure 1). They had clear, vesicular, or mottled chromatin centered by one or more nucleoli. “Hodgkin-like” cells were observed in 65% of cases (32/49, 65%) without Reed–Sternberg cells. In some cases, the leukocyte population was polymorphic, with lymphoma cells being associated with plasma cells (13/49, 26%), histiocytes (12/49, 24%), or eosinophils (8/49, 16%). A granulomatous background with histiocytes and giant cells was observed in two cases (2/33, 6%).

### 3.3. Immunohistochemistry Results

**Figure 2:**
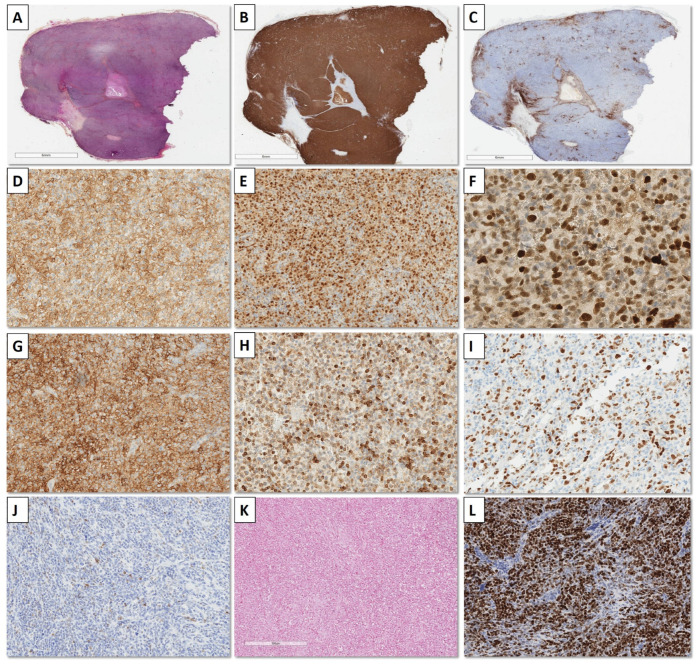
Immunohistochemistry of one PMBL case (case n°26). (A) A HES section showing a pseudo-nodular proliferation; (**B**) (CD20, ×2.5) lymphomatous cells expressed pan-B markers; (**C**) (CD3, ×2.5): CD3 staining highlighting associated reactional T-cells of the background; (**D**) (CD30, ×20): CD30 was expressed with partial a heterogeneous staining; (**E**) (BCL6, ×20) and (**F**) (MUM1, ×10): B-cells presented an activated B-cells phenotype with expression of BCL6 and MUM1, without CD10 expression; (**G**) (CD23, ×20): CD23 was expressed with a diffuse and intense pattern; (**H**) (BCL2, ×20): BCL2 was overexpressed on 60% of B-cells; (**I**) (MYC, ×20): MYC was expressed on less than 40% of B-cells; (**J**) (P53, ×20): there was no P53 overexpression: (**K**) (HIS EBERs, ×10): in situ hybridization against EBER RNAs was negative; (**L**) (Ki-67/MIB-1, ×10): proliferative index using Ki-67 was evaluated at 80%.

The phenotypic profile of the 49 PMBL cases is summarized in Table 2 and illustrated in Figure 2. Most patients presented a non-germinal center phenotype (non-GC-B) using the Hans algorithm (43/49, 88%), except for six cases, of which four were stained only with BCL6 (4/49, 8%), and two were stained with BCL6 and CD10 (2/49, 4%). EBER-ISH was positive in two cases (4%) and confirmed using LMP1 immunohistochemistry with 30 and 60% infected cells. These two EBER+ cases have been added as illustrations in the Supplementary Materials Data of the paper.

### 3.4. Fluorescence In Situ Hybridization (FISH) Results

FISH techniques were performed in cases with enough material and acceptable cell-crush artifacts. FISH analysis of MYC, BCL2, BCL6, and CIITA, respectively, were performed in 19/49 (39%), 10/49 (20%), 10/49 (20%), and 19/49 (39%) cases. MYC was rearranged in 1/19 (5%), BCL6 in 1/10 (10%), and CIITA in 10/31 (32%) patients. None of the cases displayed BCL2 rearrangement.

### 3.5. Mutational Characteristics by Targeted Sequencing

Among the 49 cases, 16 were excluded from the TNGS analysis (seven due to material exhaustion and nine because the material was no longer available).

**Figure 3:**
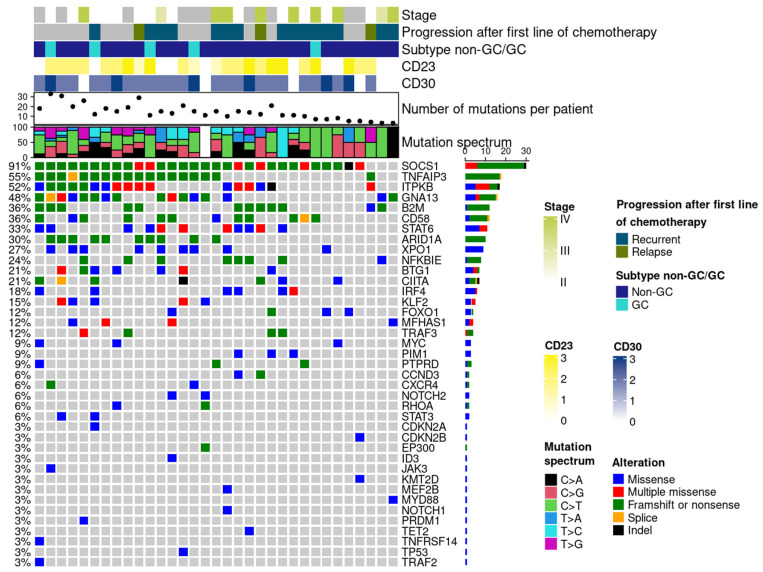
Mutational landscape of PMBL tumors. (**Bottom**) Oncoplot showing the somatic mutational landscape of PMBL. All mutated genes are shown. On the left, the frequency of mutation is indicated for each gene. Bar plots at the right represent the percentage of mutation alterations. (**Above**) Plots showing the mutational spectrum of PMBL. Plot showing the number of mutations per patient. (**Top**) Bar plots represent the positive cases for each marker (CD30, CD23), the GC/non-GC subtypes, the progression after chemotherapy, and the tumor stage.

NGS analysis was performed on 33 cases and revealed that all patients had at least one mutation in any of the 40 genes in the targeted NGS approach. The median frequency of mutations per patient was 13 (range: 3–33). The average depth was 4432.63 (ranging from 257 to 27,313). The average VAF was 26.6% (5.2 to 84.7%) [32]. The distribution of mutations found in the PMBL cohort is shown in Figure 3. The most frequent pathogenic variants were: *SOCS1* (30/33, 91%), *TNFAIP3* (18/33, 54.5%), *ITPKB* (17/33, 51.5%), *GNA13* (16/33, 48.5%), *CD58* (12/33, 36.4%), *B2M* (12/33; 36.4%), *STAT6* (11/33, 33.3%), and *ARID1A* (10/33, 30.3%), *XPO1* (9/33, 27.3%), *CIITA* (8/33, 24%), and *NFKBIE* (8/33, 24%).

Focusing on *SOCS1* pathogenic variants, only variants with a VAF of at least 5% were reported [29,32]. The median frequency of *SOCS1* mutations per patient was 5.45 (range: 1–17). The average depth was 4446.25 (ranging from 257 to 11,275). The average VAF was 24.5% (ranging from 5 to 71.8%). All mutations occurred on exon 2, mainly missense (119/180, 66%) or frameshift (27/180, 15%). Mutations retained in the report were: (i) class 3: variant of uncertain clinical significance (74/180, 41%); (ii) class 4: likely pathogenic (70/180, 39%); or (iii) class 5: pathogenic (36/180, 20%). G/C nucleotides were more frequently targeted (45/180, 25%) [33]. The detailed list of *SOCS1* variants is available in the Supplemental Data (Supplementary Materials Table S2).

All variants of *XPO1* involved the recurrent codon 571 variant (p.E571K).

**Figure 4:**
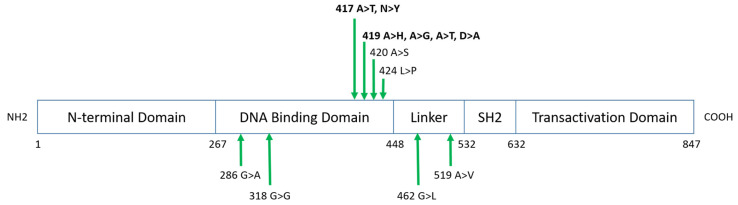
Schema of the STAT6 protein domain structure. The approximate location of somatic mutations identified in *STAT6* is indicated.

Focusing on *STAT6* mutations, the median frequency per patient was 1,45 (range 1–2). The average depth was 4355 (ranging from 1054 to 6986). The average VAF was 24.8% (ranging from 14 to 83.6%). All were missense mutations. The hotspot was located both in amino acid residue 417 (5/16, 31%) and 419 (5/16, 31%) (Figure 4), which are part of the *STAT6* DNA binding domain, and located along the surface of the protein at the DNA–protein interface [34]. Going further, 87.5% of *STAT6* mutations were found on the DNA binding domain. Co-mutations of *SOCS1* and *STAT6* occurred in 11/33 (33%) cases. The expression of CD23 was not significantly different according to *STAT6* mutations (*p* = 0.13). Using the Pearson correlation, there was no correlation between CD23 expression and *STAT6* mutations (R = 0.303, IC95[−0.045; 0.5854]).

## 4. Discussion

This study describes PMBL from a histological and molecular point of view, allowing pathologists to recognize this entity at diagnosis.

The follow-up data were only available for 25/49 patients (51%). The samples from the remaining 24 patients were external cases (samples sent by pathologists for an expert review to our pathology department) [1]. These patients received treatment in another hospital, so the clinical and follow-up data were sometimes incomplete. Most patients were treated using an R-ACVBP regimen (19/25, 76%), followed by a subsequent sequential consolidation (two cycles of methotrexate, four cycles of rituximab with etoposide and ifosfamide and two cycles of cytarabine) (17/19, 90%), or autologous stem cell transplant in two cases (2/19, 10%). The end-of-treatment complete metabolic response rate was 84%, which agrees with other studies [35]. Among the four patients who relapsed, two were treated using the R-DA-EPOCH regimen, and two using the R-ACVBP regimen. These four patients are in complete remission (follow-up from 26 to 47 months). Three received CAR-T-cell therapy; the last received another line of chemotherapy using brentuximab-vedotin and pembrolizumab [18].

The slight female predominance, the median age, and the predominance of stage II disease are in agreement with previous studies [36]. However, some findings slightly contrast with the literature data. PMBL are reported to display a non-GC-B phenotype classically, but six cases from the present study presented a GC-B phenotype indeed, according to Hans’s algorithm. Among them, four did not express MUM1 (4/49, 8%), and two were stained with BCL6 and CD10 (2/49, 4%). MUM1 expression is classical but inconstant in PMBL [37], and some authors suggested that MUM1 expression may inversely affect overall survival [37]. Expression of CD10 (20%), BCL6 (50–60%), and MUM1 (40–70%) is described as variables in PMBL [8,37], illustrating the difficulties in classifying these entities. In particular, in cases expressing both CD10 and BCL6, the differential diagnosis with follicular lymphoma or GC-B-diffuse large B-cell lymphoma should be raised, and molecular techniques must support the diagnosis. PMBL are also described as almost always negative for Epstein–Barr virus (EBV). However, such exceptions have already been described [7,38,39], and the two cases in the present study were otherwise typical of PMBL (mediastinal mass, specific morphological aspect, diffuse expression of B-cell markers, non-GCB phenotype, CD30+partial, CD23+partial/focal, and with *CIITA* rearrangement in FISH).

The present study also describes the results of FISH techniques and highlights the difficulties encountered in daily practice. Indeed, FISH techniques could not be performed in cases with significant cell-crush artifacts or insufficient material, reducing the number of patients included. These problems are often encountered in PMBL due to the difficulty of biopsying a mediastinal mass and cell-crushing artifacts linked to the specific histological characteristics of PMBL. Concerning cytogenetics, according to data from the literature, *CIITA* breaks are recurrent in PMBL (38–53%) [40]. They were present in 32% of cases in the present study (10/31, 32%). *CIITA* rearrangement has been described as significantly associated with shorter disease-specific survival rates [40]. These data could not be confirmed in this study, which was not designed to correlate histomolecular findings with prognosis. For information, among the eight patients with *CIITA* break with known evolution, two presented an unfavorable evolution, requiring CAR-T cell therapy. *CMYC*, *BCL2*, and *BCL6* rearrangements are rarely described in PMBL [41,42,43]. In the present study, one case displayed *BCL6* and CMYC rearrangements [44]. This case presented a molecular profile characteristic of PMBL (*SOCS1*, *B2M*, *CD58*, *CIITA*, *ITPKB*, *STAT3*, and *TET2* mutations). Double-hit PMBL are rarely reported in the literature [45,46]. After the exclusion of an IGH rearrangement of *MYC* using a MYC: IGH fusion probe (Vysis IGH/MYC/CEP 8 Tri-Color Dual Fusion probe), these rearrangements of *MYC* and *B*CL6 may be explained by a reciprocal t(3;8)(3q27; 8q24) *BCL6*/*MYC* translocation. Data on the clinical course of this patient are not available. In the literature, FISH studies have also demonstrated rearrangements at the *PDL1* locus (9p24.1) that are specific to PMBL and correlate with PDL1 expression using immunohistochemistry [41].

Molecular biology can help the pathologist to confirm the diagnosis of PMBL. The most frequent pathogenic variants were *SOCS1* (30/33, 91%), *TNFAIP3* (18/33, 54.5%), *ITPKB* (17/33, 51.5%), *GNA13* (16/33, 53.3%), *CD58* (13/33, 48.5%), *XPO1* (9/33, 37.3%), *CIITA* (9/33, 37.3%), *B2M* (12/33, 36.4%), *STAT6* (11/33, 33.3%), and *ARID1A* (10/33, 30.3%). These results are also correlated with those from the literature where PMBL often presents mutations of *SOCS1* (60%), *STAT6* (45%), *XPO1* (30%), and *PTPN1* (20%), leading to constitutive activation of the JAK/STAT pathway [3,47,48]. They are associated with mutations of *TNFAIP3/A20* (30–60%), *ITPKB* (40–50%), *NFKBIE* (30%), *IL4R* (30%), *NFKB2* (10%), or *IKBKB* (10%), leading to activation of the NF-κB pathway [3]. Mutations in genes involved in interferon signaling have also been described, including *IRF2BP2* (22%), *IRF8* (12%), *IRF4* (11%), and *CISH* (10%), as well as mutations involved in the immune escape, such as *B2M* (45%), *CIITA,* and *CD58* (20%) [47,49].

The present study describes mutations of *STAT6* in 33% of the cases, which is in accordance with the literature (36%) [50], and *SOCS1* mutations in 91% of cases, which is higher than that reported (45% to 60%) in the literature [51,52,53]. *SOCS1* and STAT6 mutations are not highly specific to PMBL [54]. They may be found in other B-cell lymphomas, i.e., classical Hodgkin lymphoma (CHL) [55,56], nodular lymphocyte-predominant Hodgkin lymphoma [54], diffuse follicular lymphoma [34,57], or other large B-cell lymphomas 52]. CHL therefore appears to be a diagnostic pitfall, due to its similar mutational profile, the most frequent mutations in CHL being: *SOCS1* (59%), *TNFAIP3* (36%), *STAT6* (32%), *B2M* (26%), *PTPN1* (20%), *GNA13* (24%), *NFKBIE* (15%), *XPO1* (18%), and *TP53* (9–17%) [48,58,59]. CHL and PMBL may be derived from the same cell of origin, a thymic B lymphocyte [60,61], which may explain overlapping disease evolution of these entities [40,62].

Constitutive activation of STAT6 can occur via three mechanisms [54]. The most classical, which reproduces the classical JAK-STAT pathway, is the phosphorylation of STAT6 via the binding of interleukin (IL) -4 or -13 to their receptors, leading to the transduction of phosphorylated-STAT6 (p-STAT6) into the nucleus. Expression of phosphorylated STAT6 without IL4/IL13 transcription has been demonstrated in PMBL [63], suggesting that STAT6 activation in these lymphomas is not due to an autocrine IL4/IL13 secretion. This second activation mechanism is indeed due to *STAT6* mutations occurring in the DNA-binding domain, facilitating nuclear residency of STAT6, independent of IL–4–induced STAT6 phosphorylation. The third mechanism has been described in Hodgkin lymphomas (classical Hodgkin lymphomas and nodular lymphocyte-predominant Hodgkin lymphomas) and PMBL, in which both high JAK2 expression and p-STAT6 were observed and attributed to *SOCS1* mutations via the disruption of a negative feedback loop [52,54,64]. The present study shows a much higher frequency, with 91% of the samples mutated, of mostly missense mutations. This high mutation rate may be explained by the highly selective inclusion of PMBL cases, which were included only after an expert hematopathologist review. The high number of variants on the *SOCS1* gene (average of 5.45 per patient) could also result from the recirculation of the same clone in the germinal center or the presence of subclones. In the context of lymphoma originating from the germinal center, such as PMBL, the *SOCS1* gene is indeed a known target of the mutagenic enzyme activation-induced deaminase (AID), which initiates somatic hypermutation (SHM) by converting cytosine (C) to uracil (U) in single-stranded DNA [65,66]. This aberrant SHM phenomenon may explain the high average number of variants of *SOCS1* in PMBL [66]. The C > T transition mutation is indeed commonly referred to as the DNA “footprint” of AID and was the second most represented among *SOCS1* mutations in this study (22/120, 18%).

The expression of cell surface molecules like CD23 is induced by STAT6 activation [67]. The present study showed no statistical correlation between CD23 expression and *STAT6* mutations. These findings have already been discussed in other lymphomas, particularly B-chronic lymphocytic leukemia [68], indicating that CD23 expression in lymphomas may be due to factors other than IL-4 or STAT6.

The present study highlights precise clinicopathological and molecular characteristics, allowing better recognition of PMBL in daily practice. A bundle of arguments will then be required to distinguish PMBL from CHL, MGZL, or DLBCL to perform an accurate diagnosis. As described before, PMBL are treated according to a particular regimen; therefore, it is essential to recognize this entity. Integrated histomolecular diagnosis is then mandatory in these entities; finding a characteristic mutational profile may help the pathologist. Improved knowledge of this entity at the molecular level could also help identify therapeutic targets. Indeed, complete remission after one line of chemotherapy seems to be the rule for most PMBL patients with PFS and OS of 77–95% and 84–98%, respectively, using R-CHOP with or without RT and PFS and OS of 93% and 97% using DA-EPOCH-R regimen [69]. Despite these promising results, approximately 10–20% of patients are refractory to this first line or present an early recurrence. Clinical trials have established PD-1 blockade for these patients as a promising treatment strategy for these lymphomas if associated with CD30 antibodies [18]. CAR-T cell therapy has also proven effective in a few studies [17].

## 5. Conclusions

The present study describes a PMBL cohort on the morphological, immunohistochemical, and molecular levels to provide pathologists with daily routine tools. These data also reinforce the interest in an integrated histomolecular diagnosis to allow precision diagnosis as early as possible and to adapt the therapeutic strategy.

## Figures and Tables

**Table 1:** Overview of the clinical characteristics, received treatments, and evolution of the 33 analyzed cases of primary mediastinal b-cell lymphomas (PMBL).

		Number (*n*)	Percentage (%)	Average	Ranges
Age (*n* = 49)				38.9	21–83
Sex (*n* = 49)	Male	23	47		
	Female	26	53		
Symptoms (*n* = 33)	Cough	10			
	Dyspnea	13			
	Chest pain	13			
	Superior vena cava syndrome	6			
Elevated LDH (N < 250 UI/L) (*n* = 25)		23	92	409	249–802
Tumor size (mm) (*n* = 25)				101	52–150
Ann Arbor (*n* = 33)	II	22	67		
	III-IV	11	33		
Chemotherapy (*n* = 25)	R-CHOP	2	8		
	R-DA-EPOCH	4	16		
	R-ACVBP	19	76		
Evolution (*n* = 25)	Complete remission	21	84		
	Relapse	4	16		

**Table 2:** Immunophenotypic features and results of EBERs in situ hybridization in the 49 analyzed primary mediastinal B-cell lymphomas (PMBL) cases. Predefined cut-offs were used for most of the antibodies except for the proliferation index with Ki-67, which benefited from precise quantitative evaluation. Labeling was considered diffuse if more than 75% of cells were labeled, partial if 30 to 75% of cells were marked, and focal if less than 30% were labeled. This scoring system was adapted for BCL2 and CMYC. BCL2 was considered positive if ≥50% of cells were stained, with a partial staining if 50 to 75% of cells were stained, or diffuse staining if >75% of cells were labeled. CMYC was considered positive when >40% of the tumor cells had nuclear staining, with a partial staining if 40 to 75% of cells were stained or a diffuse staining if >75% of cells were labeled. CD10, BCL6, and MUM1 were defined as positive if at least 30% of neoplastic cells were stained, according to the Hans algorithm for DLBCL.

Expression	CD20	CD10	BCL6	CD30	CD23	CD15	BCL2	MUM1	MYC	P63	PDL1	EBER-ISH
Diffuse (*n*, (%))	49 (100%)		39 (79%)	10 (20%)	11 (22%)		29 (59%)	29 (59%)	15 (31%)	2 (6%)	2 (7%)	
Partial (*n*, (%))		2 (4%)	8 (16%)	33 (67%)	22 (45%)		2 (4%)	14 (28%)		7 (23%)	13 (45%)	2 (4%)
Focal (*n*, (%))			1 (2%)		4 (8%)		3 (6%)		34 (69%)	7 (23%)	12 (41%)	
Negative (*n*, (%))		47 (96%)	1 (2%)	6 (12%)	12 (24%)	33 (100%)	15 (31%)	6 (12%)		15 (48%)	2 (7%)	47 (96%)

## Data Availability

Data are available on demand.

## Supplementary Materials

The following supporting information can be downloaded at: https://www.mdpi.com/article/10.3390/cancers15194866/s1, Figure S1: Histological aspects of EBV+ case n°1; Figure S2: Histological aspects of EBV+ case n°2; Table S1: References of antibodies used for immunohistochemistry; Table S2: Detailed list of *SOCS1* variants.
