# Supplementary material for: Molecular Characterization of Primary Mediastinal Large B-Cell Lymphomas
Source: Cancers (Basel). 2023 Oct 6;15(19):4866. doi: 10.3390/cancers15194866 (PMC10571533; doi:10.3390/cancers15194866)
Supplement: Supplementary file 1 [file cancers-15-04866-s001.zip › Supplemental Figures 1 et 2.pptx]

## Slide 1
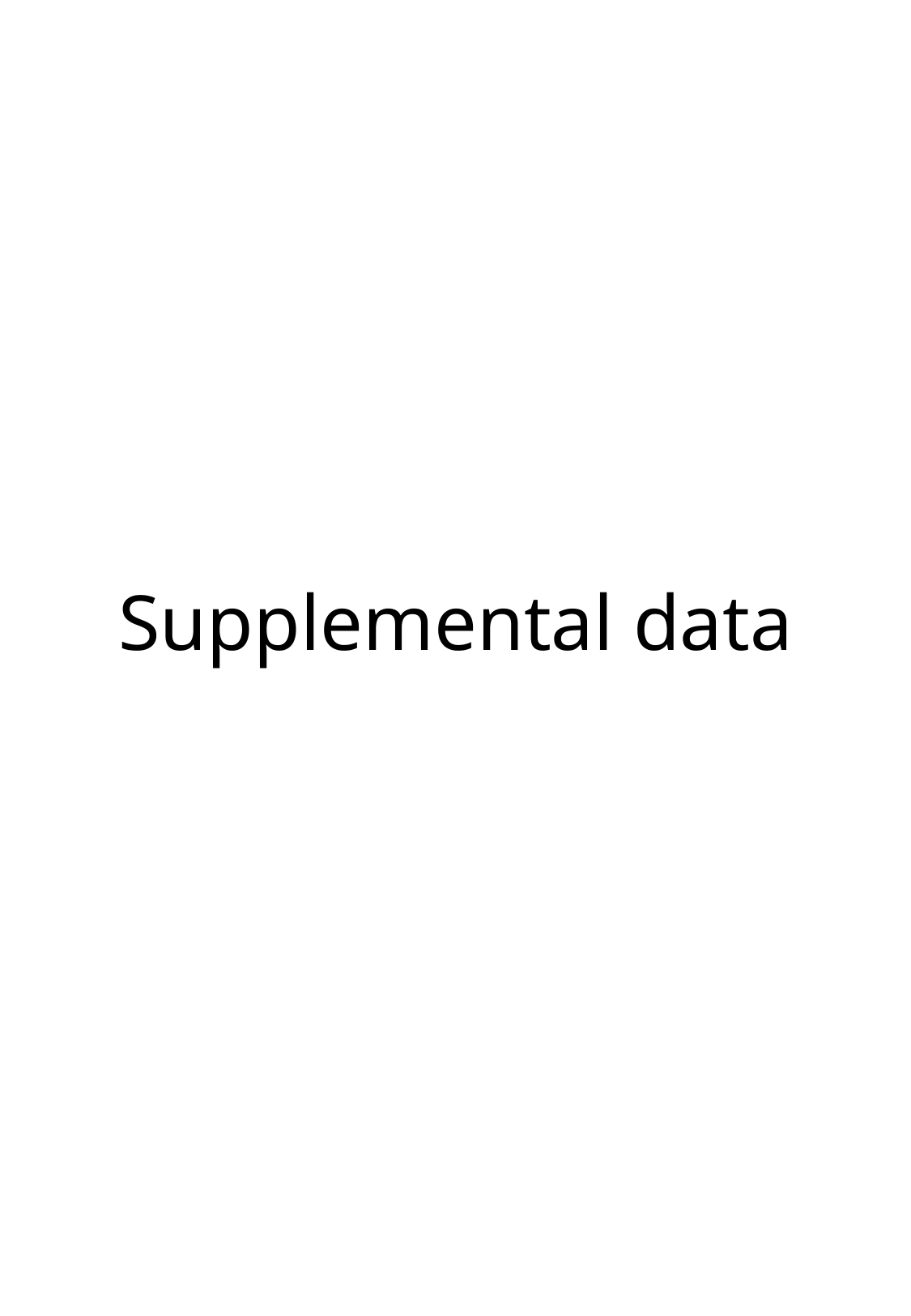

# Supplemental data

## Slide 2
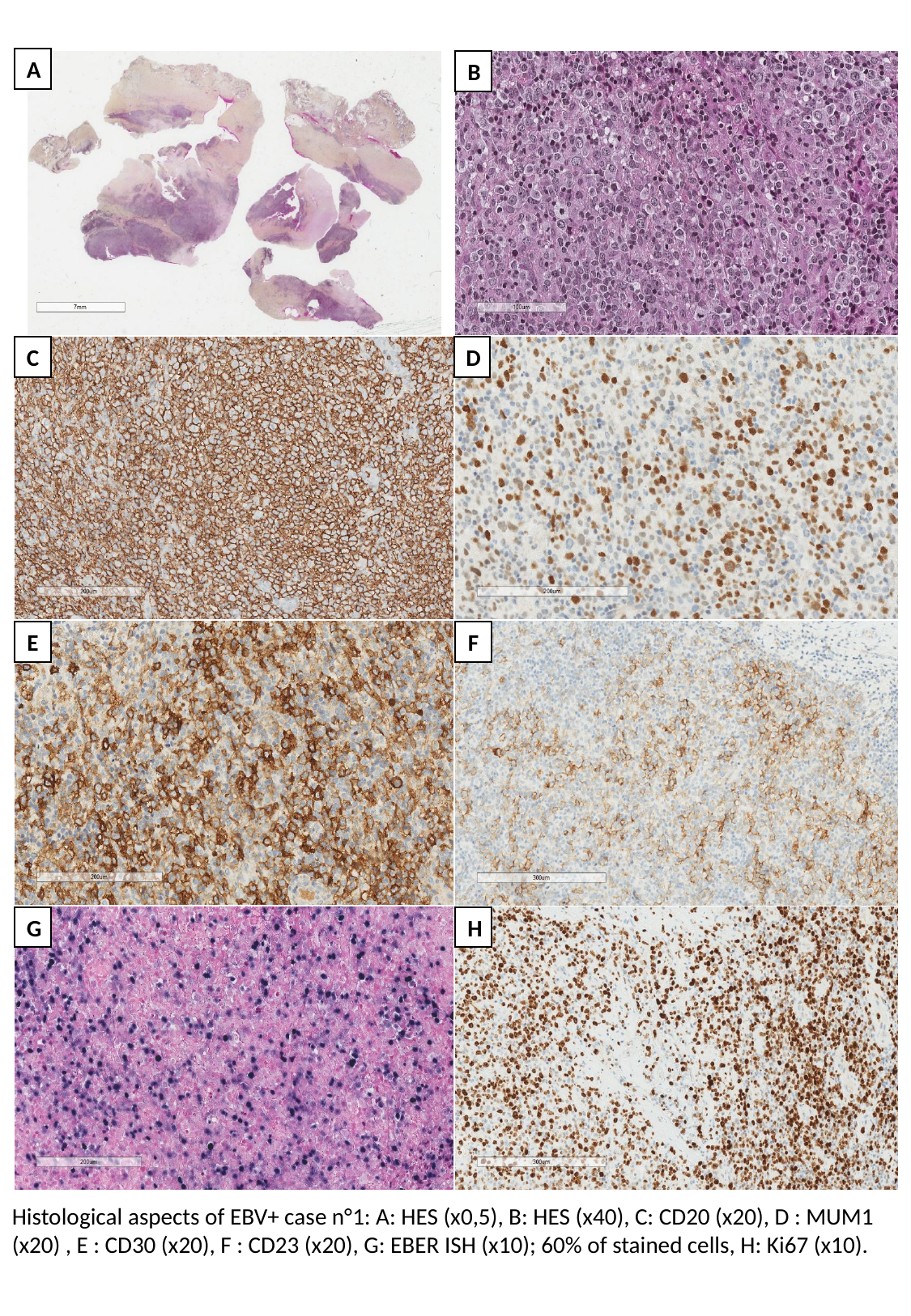

A
B
C
D
E
F
G
H
Histological aspects of EBV+ case n°1: A: HES (x0,5), B: HES (x40), C: CD20 (x20), D : MUM1 (x20) , E : CD30 (x20), F : CD23 (x20), G: EBER ISH (x10); 60% of stained cells, H: Ki67 (x10).

## Slide 3
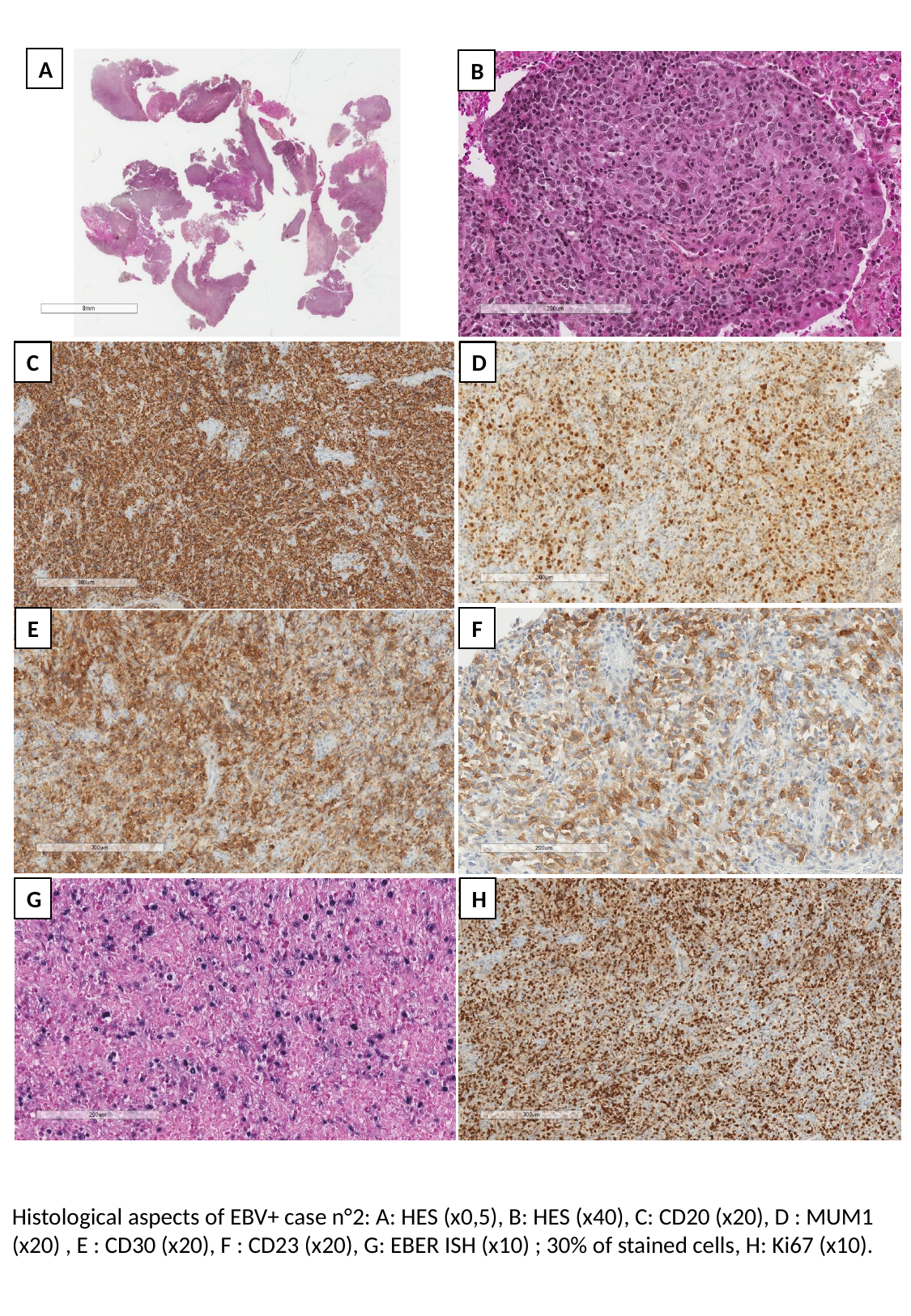

A
B
C
D
F
E
G
H
Histological aspects of EBV+ case n°2: A: HES (x0,5), B: HES (x40), C: CD20 (x20), D : MUM1 (x20) , E : CD30 (x20), F : CD23 (x20), G: EBER ISH (x10) ; 30% of stained cells, H: Ki67 (x10).
