# Supplementary material for: Molecular Characterization of Primary Mediastinal Large B-Cell Lymphomas
Source: Cancers (Basel). 2023 Oct 6;15(19):4866. doi: 10.3390/cancers15194866 (PMC10571533; doi:10.3390/cancers15194866)
Supplement: Supplementary file 1 [file cancers-15-04866-s001.zip › Supplemental Materiel and methods and Suppl Table S1.docx]

**Supplemental Materiel and methods :**

**Immunohistochemistry**

Three μm-thick tissue sections were cut from paraffin-embedded skin biopsy, dried, de-waxed, rehydrated and slides were unmasked with ER1 Solution (Leica, Bond Epitope Retrieval Solution 1). Detailed references of used antibodies are available in supplemental data (suppl. Table 1). Immunochemistry was performed using standard procedures in an automated Immunostainer (Leica BOND-MAX, LEICA MICROSYSTEMES SA, NANTERRE, France ). In situ hybridization for Epstein-Barr virus (EBV) was performed on deparaffinized tissue sections using a fluorescein isothiocyanatecoupled specific peptidic nucleic acid probe (same Immunostainer).

| Antibodies/*In situ* hybridization | References |
| --- | --- |
| Anti-CD20 | Mouse monoclonal primary antibody, L26 ; Agilent Dako, Boston, USA |
| Anti-CD3 | Rabbit polyclonal primary antibody, A0452 ; Agilent Dako, Boston, USA |
| Anti-CD10 | Rabbit monoclonal primary antibody, SP67; Roche Diagnostics; Meylan; France |
| Anti-BCL6 | Mouse monoclonal primary antibody, GI191E/A8 ; Roche Diagnostics; Meylan; France |
| Anti-CD30 | Mouse monoclonal primary antibody, M0751 ; Agilent Dako, Boston, USA |
| Anti-CD15 | Mouse monoclonal primary antibody, MMA; Roche Diagnostics; Meylan; France |
| Anti-MUM1 | Rabbit monoclonal primary antibody, MRQ43; Merck; 1:200; Saint-Quentin Fallavier; France |
| Anti-BCL2 | Mouse monoclonal primary antibody, 124; Roche Diagnostics; Meylan; France |
| Anti-CMYC | Rabbit monoclonal primary antibody, Y69; Roche Diagnostics; Meylan; France |
| Anti-CD23 | Rabbit monoclonal primary antibody, SP23 ; Thermofisher, Waltham, US |
| Ki67/MIB1 | Mouse monoclonal primary antibody, M7240; Agilent Dako, Boston, USA |
| HIS EBV encoded RNAs | 800-2842; Roche Diagnostics, Ventana; Medical Systems; Mannheim; Allemagne |
| Anti-LMP1 | Mouse monoclonal primary antibody, M0897 ; Agilent Dako, Boston, USA |

**Supplemental Table S1**: **References of antibodies used for immunohistochemistry.**
